# Exploring access to end of life care for ethnic minorities with end stage kidney disease through recruitment in action research

**DOI:** 10.1186/s12904-016-0128-1

**Published:** 2016-07-11

**Authors:** Emma Wilkinson, Gurch Randhawa, Edwina Brown, Maria Da Silva Gane, John Stoves, Graham Warwick, Tahira Akhtar, Regina Magee, Sue Sharman, Ken Farrington

**Affiliations:** Institute for Health Research, University of Bedfordshire, Luton, UK; Imperial College Healthcare NHS Trust, London, UK; East and North Herts NHS Trust, Stevenage, UK; Bradford Teaching Hospitals NHS Foundation Trust, Bradford, UK; University Hospitals of Leicester NHS Trust, Leicester, UK

**Keywords:** Access, Ethnicity, End of life care, Action research, Communication, End stage kidney disease

## Abstract

**Background:**

Variation in provision of palliative care in kidney services and practitioner concerns to provide equitable access led to the development of this study which focussed on the perspectives of South Asian patients and their care providers. As people with a South Asian background experience a higher risk of Type 2 Diabetes (T2DM) and end stage kidney failure (ESKF) compared to the majority population but wait longer for a transplant, there is a need for end of life care to be accessible for this group of patients. Furthermore because non English speakers and people at end of life are often excluded from research there is a dearth of research evidence with which to inform service improvement. This paper aims to explore issues relating to the process of recruitment of patients for a research project which contribute to our understanding of access to end of life care for ethnic minority patients in the kidney setting.

**Methods:**

The study employed an action research methodology with interviews and focus groups to capture and reflect on the process of engaging with South Asian patients about end of life care. Researchers and kidney care clinicians on four NHS sites in the UK recruited South Asian patients with ESKF who were requiring end of life care to take part in individual interviews; and other clinicians who provided care to South Asian kidney patients at end of life to take part in focus groups exploring end of life care issues. In action research planning, action and evaluation are interlinked and data were analysed with emergent themes fed back to care providers through the research cycle. Reflections on the process of patient recruitment generated focus group discussions about access which were analysed thematically and reported here.

**Results:**

Sixteen patients were recruited to interview and 45 different care providers took part in 14 focus groups across the sites. The process of recruiting patients to interview and subsequent focus group data highlighted some of the key issues concerning access to end of life care. These were: the identification of patients approaching end of life; and their awareness of end of life care; language barriers and informal carers’ roles in mediating communication; and contrasting cultures in end of life kidney care.

**Conclusions:**

Reflection on the process of recruitment in this action research study provided insight into the complex scenario of end of life in kidney care. Some of the emerging issues such as the difficulty identifying patients are likely to be common across all patient groups, whilst others concerning language barriers and third party communication are more specific to ethnic minorities. A focus on South Asian ethnicity contributes to better understanding of patient perspectives and generic concepts as well as access to end of life kidney care for this group of patients in the UK. Action research was a useful methodology for achieving this and for informing future research to include informal carers and other ethnic groups.

## Background

The global impact on ageing societies living and dying with the increasing burden of non-communicable diseases like Type 2 Diabetes Mellitus (T2DM) and complications is predicted to great in the future [1]. Although South Asian populations (those originating from the Indian subcontinent – India, Pakistan, Bangladesh and Sri Lanka) in the UK are relatively young compared to the White European population they are ageing and - with higher prevalence of T2DM -related end stage kidney failure (ESRF), relative risk of acceptance rate to renal replacement therapy (RRT) of 5.8 compared to the White European population [2], and longer waiting times for donor organs [3] - there is a demand for end of life care which meets this group’s needs.

As well as shifts in the age structure of populations, many countries are becoming increasingly diverse as populations grow and migration patterns respond to economic and political change around the world. By the 2050s, ethnic minorities are predicted to make up 30–40% of the UK population [4] so there is a growing need for health services to be commissioned for a diverse population. This is the case elsewhere in the world too and inequalities associated with migration and socioeconomic status can contribute to inequalities in access to healthcare through generations [5].

Inequalities in outcomes and access to services have been documented as common experiences for minority ethnic groups in the UK [6]. Research investigating access to care for ethnic minority groups with life limiting conditions and ethnic patient groups [7, 8] have found that patients who have non cancer conditions and experience cultural and language barriers in communication with care providers are particularly at risk of inadequate end of life care. Furthermore a survey of UK kidney services in 2005 found little evidence of palliative care and that it was variable across the country [9].

Policy and practice have moved on during the intervening years [10] with guidance for kidney services to meet the needs of their patients requiring end of life care [11]. However, much of the research about quality and patient experience of end of life care in the kidney setting has been conducted with English speaking patients only [12]. This is a limitation for producing guidance for culturally competent kidney care in the UK where there are established South Asian communities with older people who may require these services but may not speak English as their first language [9, 13] and experience other cultural barriers.

This research aimed to explore end of life care for South Asian patients with ESRF and in this paper we reflect on the process of identifying and recruiting patients who were in or near the end of life phase of kidney care. Whilst the issues articulated were specific to the experiences of individual participants they are discussed in the context of end of life care in the kidney setting and a concept of cultural competency which considers the heterogeneity and similarities found within and between different population groups.

## Methods

### Design

An action research methodology was used to explore end of life care for South Asian patients with kidney disease to better understand inequalities in access and experience of end of life care and how these can be reduced.

**Fig. 1:**
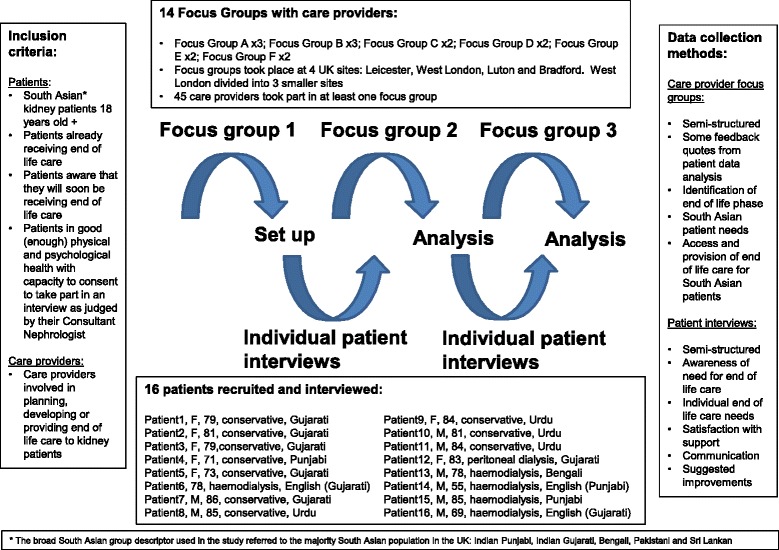
Action research and recruitment

Action research is a collaborative research methodology which enables researchers and practitioners to work together to explore research questions in relation to theory and practice [14, 15]. It operates in a series of research cycles of action and reflection, drawing on the process of conducting the research as well as research outcomes to generate knowledge and understanding of the issues. The main characteristics of action research are that it seeks to be participatory, democratic and to improve practice. In this exploratory study the first cycles of action towards understanding access for service improvement for South Asian patients was for researchers and those providing end of life care to diverse populations to come together to explore the issues, to include the patient voice through interviews with patients about their experiences and further reflection on the issues in relation to practice (Fig. 1).

### Inclusion criteria

This project purposively focussed on South Asian patients with kidney disease to inform delivery of care across diverse patient populations and was located at NHS sites in the UK which serve local areas where South Asian communities make up a significant proportion (40–50 %) of the population: Leicester, Luton, Bradford and parts of West London [16, 17]. Kidney clinicians with an interest in end of life care were recruited as principal investigators (PI) at the four sites.

Patient participants were recruited via the local PI and team, based on their assessment of individual patient need for end of life care, to take part in a single interview. The patient inclusion criteria was: South Asian renal patients (aged 18 years old and upwards) either already in receipt of end-of-life care or South Asian renal patients who are already aware that they will soon be receiving end-of-life care and are judged by their Consultant nephrologist to be in good physical and psychological health with capacity to consent. Care provider participants were recruited, also by the PI, to take part in up to three focus groups (see Fig. 1). The care provider participant inclusion criteria were: care providers involved with planning, developing or providing end-of-life care to renal patients.

### Interview guide

Both individual interview and focus group were semi-structured. The interview schedule had questions and prompts to guide the participants’ account of their experiences: awareness of the need for end of life care; individual end of life care needs; satisfaction with support; communication; and suggested improvements to services. The focus group guide provided a combination of feedback quotes from patient and focus group data analysis together with questions covering how the end of life phase was identified; South Asian patient needs; access and provision of end of life care for South Asian patients. These qualitative data collection methods were set within an action research design which provided a framework for the research design and implementation (Fig. 1).

### Data collection

Informed consent was taken from participants prior to each interview or focus group which was audio recorded. Both individual patient interviews and care provider focus groups were a maximum one hour in duration. Patient interviews were conducted in the participant’s preferred spoken language and location, which was (in almost all cases) their home. Bilingual interviewers carried out the patient interviews in South Asian languages (Urdu, Punjabi, Gujarati and Bengali) or they were conducted in English by the lead researcher who also facilitated the focus groups. To ensure quality and trustworthiness of the data, interviewers were trained and supported over the study timeframe through a series of workshops which focussed on the linguistic and contextual meanings within the interview schedule as well as ethical issues of conducting sensitive interviews.

### Analysis

Recordings were transcribed verbatim and transcripts analysed thematically [18]. Transcripts were read and re-read by the lead researcher, an experienced qualitative researcher, and broad themes identified through comparison of their content. Emerging themes with example quotes were fed back to the focus groups with care providers for focus group discussion and to help with interpretation. Furthermore a joint site meeting of PIs with representation from care providers at each participating site took place towards the end of the research to explore the findings as a group; and the chief investigator of the research had oversight of the whole data analysis process.

### Ethical approval

The research had been approved by the National Research Ethics Service (Essex 1 Research Ethics Committee, ref no: 09/H0301/62) prior to commencement and interviews were carried out in accordance with the approved protocol and approved interview paperwork.

## Results

This paper reports on the themes emerging from the data concerning the recruitment of patients which was an important aspect of the research. To maintain contextual integrity we present our analysis of the data, which was predominantly care provider data with some patient data where appropriate, as broad themes with selected quotes to illustrate meaning.

Sixteen patients were recruited to the study (10 conservative care and 6 dialysis); and 8 were female and 8 male with a median patient age of 80 years old as reported previously [19].

Forty-five different care providers took part in at least one of 14 focus groups that took place across the four sites as part of the action research cycle (Fig. 1). For pragmatic reasons of conducting the focus groups with NHS staff from different hospital sites and because of the size and organisation of the West London kidney services, the West London site was split into three for the purpose of data collection (Fig. 1).

The variety of roles represented by focus group participants were: Senior nurse nephrology ward, Head nurse dialysis unit, Renal counsellor, Renal social worker, Consultant nephrologist, Dialysis nurse, Social practitioner, Renal liaison sister, Staff nurse, Renal clinic sister, Consultant in palliative care, Registrar in palliative care, Home care team nurse, Renal pharmacist, Research nurse, Renal community sister, Deputy sister renal ward, Renal community nurse, Peritoneal dialysis sister, Senior clinical nurse manager peritioneal dialysis, Senior research nurse, Research nurse end of life care, Head nurse renal unit, Clinical practice educator for renal services, Ethnic minority support worker. These reflected some of the different care scenarios that people with ESRF experience i.e. in the hospital setting: haemodialysis, peritoneal dialysis, acute ward and outpatient clinics; and in the community: palliative care and home care teams. Less than half the patient sample, 6 out of 16 patients, was recruited from haemodialysis or peritoneal dialysis units.

### Identifying patients with end of life care needs

Care providers articulated the difficulty in identifying when patients are reaching the end of life phase as patients, particularly those on dialysis, were described as the ‘survivors’ of their cohort and as ‘bouncing back’ and having ‘nine lives’. Care providers used different working definitions of end of life which influenced when discussions about end of life care and advance care planning took place.

*‘And sometimes within the renal team there can be inconsistencies of people’s perception about the end of life issue because renal patients bounce back so many times, erm, particularly say in the haemodialysis patient population that we see, they’ve had an acute event and people might just see that acute event in isolation and they’ve had several admissions into hospital and it would seem, in discussion with some of my colleagues, that this doesn’t necessarily put the bits of the puzzle together to have the whole picture and they bounce back in several times but no one has broached the subject of well actually this is a sign that you are in the deteriorating phase of your long term illness and that we need to make some decisions and possibly people avoid talking about it. Erm, and whether it’s because they think it’s taboo or it’s just their - it’s the way that they work, not everybody openly wants to talk about it. But that’s healthcare professionals as much as the patients and that can influence when and if it gets spoken about in advance.’*

*(Care provider 2, FGE1)*

*‘You know, having discussions with patients, with family members, whether they’re aware of that prognosis, I would definitely say yes, that is part of end of life care and that can happen at any time. I suppose it’s that definition, ‘end of life care’.’*

*(Care provider 2, FGA1)*

It was suggested that the patients included in the research sample might be atypical because they had engaged in a conversation, however brief, about end of life care, whereas the majority of South Asian patients, who might have fulfilled the criterion of soon to be requiring end of life care, probably would not have.

*‘The patients who will not engage in the study are the ones you really want to talk to. That’s the issue. So what you’re going to end up doing is talking to the atypical patient…the patient who was engaged in an end of life discussion…who has agreed to an end of life pathway……and is articulate enough to talk to you. And the other 99 % of the patients will not be eligible for the study because they won’t agree to end of life care,….or if they have, they can’t speak to, they’re not articulate enough to get into that conversation, or they won’t…..it’s not a cultural thing apart from the language. My gut feeling is it’s not a cultural thing but an educational thing…and that if you are more educated you value quality of life and you understand the concept of life expectancy, whether you are White, Black or South Asian and if you are not so educated you want the most of life you possibly extract, in a simplistic sense, erm, because that’s your lot in life. That’s my take on it but it’s not tested….’*

*(Care provider 3, FGB1)*

### Awareness of end of life care

Interviews with patients found that notwithstanding informed consent, a quarter of patient participants had been unaware of their identified need for end of life care which accompanied a lack of awareness of what end of life care referred to in relation to their ESRF. There was a range of understanding and awareness of end of life care.

*‘End-of-life effect is everything. I have problems, diabetic, high blood pressure, kidney. I have lots of illness… This is end-of-life going on. I am on my eighty-sixth year, so now I only have one wish from God and that is if he I am taken from here I am happy. But I do not have wishes to live for more days.’*

*(Patient 7, Male, 86, conservative care)*

*‘A person can only demand what he knows is provided. I don’t know what services are provided or what is needed. Maybe there are some good things on offer but I don’t know what they might be so I cannot say. I don’t know if this is helpful for you but I don’t want to talk about something that I know nothing about, I think that would be wrong.’*

*(Patient 8, Male, 84, conservative care patient)*

*‘I had misunderstood because I thought the kidneys were only 20 % not working. But then Dr xxxx told me that it was actually 80 % not working………. So these people came and they told me that there is nothing that can be done about the kidneys, but we will see afterwards. It took 4–6 months for me to understand this, I didn’t really understand it before. So this is the situation.’*

*(Patient 10, Male, 81, conservative patient)*

### Communication with patients and family members about end of life care

At three of the four sites roles allied to nursing: an Ethnic Minority Support Worker, Renal Research Nurse and End of Life Care Research Nurse facilitated the recruitment of patients by helping clinicians identify patients, providing clarification about the research and acting as a link to the bilingual interview team. The fourth site had no additional support role for the research and no patients were recruited. Explanations for this were the gate keeping role of family and the difficulty communicating with patients and their relatives about end of life care.

*‘It was more the family didn’t want to have that discussion and were, er, almost accusing this team: ‘so actually you’re saying my parent is dying but I’m not prepared to accept that’*

*(Care provider 1 FGC2)*

*‘…… I think it’s an unwillingness to accept; I think – from my point of view what I – it seems to be that if they discuss the end of life with their parent, say, it’s actually accepting that they will give up on their parents and they feel that it’s not an acceptable thing to do.......you know, they have to fight to the very end to keep their parent alive, they’re there at the time and I think it’s the unwillingness of even accepting that idea.’*

*(Care provider 2 FGC2)*

### Cultures within kidney care

Different nursing cultures within kidney services where communication about end of life care was inhibited or supported were described. These were broadly associated with the different treatment pathways: haemodialysis compared to peritoneal dialysis and conservative care respectively, and related to the lack of time available for discussion, lack of confidence of nurses to have conversations which touched on end of life issues and perceptions of palliative and supportive care.

*‘I’ve heard (dialysis) nurses say that they try their best not to talk to patients in much detail because they know that if they do that for ten minutes then that delays their next job by ten minutes, which is awful but, you know, you can see it from their side, their point of view, that means somebody else is ten minutes late going on and that patient would be really annoyed with them because they’d been an extra ten minutes in the waiting room. And you can just imagine that if it was a conversation that was – they knew was going to be difficult and time consuming, they wanted to give that patient the time, that it might just be easier to delay that because they’ve got so many other things to do.’*

*(Care provider 6, FGA3)*

*‘We don’t feel how I say it, happy or comfortable to go and approach the patient and ask because you always think it’s best to come up from a doctor and usually its discussed with the family sitting around and discussing with their you know their diagnosis and their other medical issues and explaining more properly than for you think you probably don’t have that knowledge to go and talk to someone …… always think it’s best to come out from a doctor.’*

*(Care provider 1 FGB1)*

*‘There's this Asian family we had just the other week, in particular the daughter went home and one of the nurses actually had to take time out and say, “look, there’s palliative care and then there’s specialist palliative care, we’re not saying your Mum’s having that we’re saying we’re getting into a support system here that, you know, you’re quite a way from the hospital, we’ll be able to get service in quicker, we’ll be able to get you those essential things where your GP says no they’re very expensive, because we can say she needs them at this point in her palliative care and get it through quicker so it can kind of open doors up.” And then they’re a bit more receptive, I would say, and had time to sort of think about well, although they recognise Mum is declining that there was a bit more acceptance to it rather than was she imminently dying.’*

*(Care provider 2, FGE1)*

## Discussion

As stated earlier, a motivation for this study was that research about end of life care in the kidney setting has mainly been conducted with English speaking patients so there is little evidence of how ethnicity might influence the needs, access and experience of end of life kidney care in the UK.

Our results suggest that from a clinical perspective identification of people with ESRF and end of life care needs can be difficult, irrespective of ethnicity. Service providers have different working definitions of end of life kidney care, so it follows there will be differences in patient experience across kidney services of when end of life care is discussed. Research in other non-cancer clinical areas [20] found similar inconsistencies suggesting that more awareness and interdisciplinary discussion of what, how, and when end of life care needs should be addressed is necessary.

In the kidney setting, testing the utility of the ‘surprise question’ (‘would you be surprised if the patient died within the next 12 months’) to predict the end of life phase with haemodialysis patients in the UK concluded that it varied according to clinical discipline, seniority and clinical setting with inter-clinician agreement improving predictive power [21]. Furthermore a prospective study of conservative care kidney patients found that notwithstanding generic trajectories for organ failure, people with kidney disease varied individually and had fluctuating individual trajectories [22]. These studies support the idea that identifying the end of life phase for people with kidney failure is not an exact science but requires knowledge of individual patients on a case by case basis.

Recruitment to this study relied on clinician assessment with patient awareness of end of life care need and the resulting sample comprised a greater number of people on a conservative care treatment plan than those receiving dialysis. This might be explained by patients on a conservative pathway having thought about or made decisions about not undergoing dialysis and therefore being more aware of end of life care than those on renal replacement therapy. Notwithstanding this and the proportion of conservative care patients in the sample, a quarter lacked awareness of end of life care or their identified need for it. The excerpts from patient interviews demonstrate the range of understanding amongst patients with ESRF and how it was associated with different factors such as their holistic view of their health, lack of awareness of services, and misunderstandings of information given during healthcare encounters.

These findings concur with previous research with a majority White kidney patient population with advanced kidney disease in Canada which found patients lacked knowledge of end of life care and palliative care, were willing to discuss end of life care but relied on their clinicians to raise the issue at the right time and had not discussed end of life care issues within the previous year [24]. The survival for older people on dialysis and conservative care is similar [23] suggesting an equal need for end of life care across different treatment pathways, however, our findings and those from other studies suggest limited access to end of life care, at least in the form of communication and advance care planning, for some South Asian patients.

Our analysis of the care provider focus groups provides insight into factors behind the lack of patient awareness of end of life care by suggesting that not all clinicians who have contact with kidney patients consider the whole picture or communicate it with patients. Reasons for this may be that they do not have time; or all the information; do not think it is appropriate or do not have the communication skills, including access to a translator if there is a language barrier. The latter, care providers suggested, was reflective of their experience of caring for South Asian kidney patients if family members were relied upon for translation which might be inaccurate, and this is a problem that has been reported elsewhere [25]. As communication with patients is part of end of life care, patients who do not speak English may miss out on early communication about their condition as well as on regular and on-going opportunities where concerns could be raised, if there is limited translation available e.g. on dialysis units.

Other researchers have drawn attention to some of these issues before, for example, the difficulties of mediated conversations in cancer care [26] and the need for advanced communication training for clinicians in the kidney setting [27]. They have highlighted the importance of understanding patient experience and the need for further research into the influence of culture on quality and access to end of life care for patients. The action research approach employed here enabled researchers to observe and discuss access issues close up with South Asian patients and their care providers through reflection on the recruitment process in this exploratory project.

Our analysis of the data from these discussions has suggested similarities between South Asian patient experience and that of White European and majority population participants in other kidney care research. Care provider data also captured a perception that it is education rather than ethnicity per se that determines access to end of life care and which is likely to be a factor across all ethnic groups. The way care providers work with the diversity of individuals within their patient population is an issue of cultural competence and this suggests that a fluid and individualised interpretation of the concept is more useful than an ethnocentric focus often found in the literature [28].

Recruitment was facilitated at three of the four sites by support workers, one of whom was bilingual, and they were an additional source of information about the research and a link to the team of bilingual research interviewers. On the site without this extra support gatekeeping by family members was a barrier to recruiting patients. Although recruitment to a research interview and access to care are different things they both require communication about what can be a sensitive topic for patients, their families and care providers. It has been suggested that the lack of participation by ethnic minority groups in research is a result of researchers not applying solutions to overcome barriers or investing necessary time and resources in recruitment rather than minority groups being ‘hard to reach’ because of their ethnicity [29]. The recruitment and interviews were achieved in this study through support workers and trained bilingual research interviewers who were able to overcome language barriers and offer a level of cultural competence in discussing end of life care with participants.

The barriers encountered with family members acting as gatekeepers and illustrated in quotes suggest that the term end of life care was synonymous for some people with death and dying and there was a wish to protect the patient from the thought that they were being ‘given up’ on. Research that has investigated the impact of clinicians discussing end of life care with patients found that rather than reducing hope participants benefitted from a realistic focus which helped to maximise support from important relationships [30]. However that finding related to a predominantly White population and the authors acknowledged that ethnicity and religion can shape individual attitudes to advance care planning. Our exploratory study together with the observations of other researchers [31] identifies a need for more research with ethnic minority patients, and importantly their families, to understand better the role of culture in access to end of life care.

Reflection on the recruitment of patients to this study revealed the influence of culture through organisational and system wide attitudes towards acknowledgment and talking about end of life. The dominant kidney specific factor with respect to this is the potential of renal replacement therapy i.e. dialysis and transplant to provide life sustaining treatment and this raises challenging decisions for patients, their families and care providers when a person approaches the end of life phase [32]. These results suggest that communication with patients about end of life issues is unlikely to take place at haemodialysis units despite the on-going and frequent contact, because the focus of care is primarily on the practical task of dialysis and there are time constraints as well as privacy issues. In addition dialysis nurses may lack the confidence to have conversations which touch on end of life care, as these are seen as the doctors’ domain to be discussed away from the unit, in clinic appointments with family present.

The lack of familiarity and understanding of a palliative approach highlighted through provider reports of talking about future care and born out through the recruitment process and patient interviews, highlights an overarching lack of awareness of the concept of end of life kidney care. Palliative care and supportive care are key elements which underpin quality end of life care and these results seem to indicate a lack of consensus amongst services and a lack of early communication with individual patients about how good end of life can be achieved.

Limitations of this research were that patient recruitment was based on clinician judgement of end of life care needs rather than on a specified definition together with their perception of patient awareness. This may have influenced the patient sample (which was less than the 30 anticipated) as well as the results and was reflected in the data that the patients recruited were likely to be atypical by having had some discussion of their life limiting condition. Not only does this suggest that access to end of life care and advance care planning is likely to be (even) less than our findings describe, it draws attention to the issue of heterogeneity within the broad South Asian ethnic grouping which questions the usefulness of ethnicity rather than culture as a concept and key determinant of access. Results obtained this way however enabled a realistic picture of understanding and communication about end of life care between care providers and patients in the kidney setting. As it was based on the recruitment criteria and process outlined above it was possible that there were other patients who were aware of their end of life care needs but who had not communicated with professionals about it.

Although this exploratory qualitative action research project does not claim generalizability it does seek to understand some of the issues and concepts concerning inequalities in access. Whilst a focus on South Asian ethnicity is relevant to the increased risk and poorer kidney outcomes [2, 3] associated with diabetes, progression of kidney disease and shortage of compatible donor organs which have genetic and physiological dimensions that relate to ethnicity, these observations on recruitment indicate it is individual and organisational culture and context in relation to awareness, understanding and communication of end of life care which are likely to be the important moderators of access.

As an early phase study, this research benefited from an action research approach which was able to draw on the experience of the variety of care providers working in kidney care. This enabled both the recruitment of patients who hitherto have not been included in research and provided insight into the research process and data collected. To take this research forward the next cycle of the research would include a broader inclusion of ethnic groups to enable a comparative element, include the participation of families and informal carers and consider the later stages of the end of life kidney care.

## Conclusions

Recruitment of South Asian patients with kidney disease by care providers to this study highlighted some of the barriers to access to end of life care in the kidney setting which care providers have to work with. These were: difficulties in identifying the end of life phase; and lack of awareness of end of life care; communication which can be influenced by family members as gatekeepers; and the different end of life kidney care scenarios.

Reflection on engaging with South Asian patients at end of life provided insight into the complex setting of end of life in kidney care and the need for future research, including with family carers, to explore the experience of South Asian patients with kidney disease in relation to culture still further.

Action research was a useful methodology for getting close to the issues raised and allowing reflection on these through the recruitment process. Clinical services need to be mindful of the diversity of the patient population that they serve and ensure that cultural competency is integral to service delivery.

## Abbreviations

ESKF, end stage kidney failure; FG, focus group; NHS, National Health Service; RRT, renal replacement therapy; T2DM, type 2 diabetes mellitus; UK, United Kingdom

## References

[1] WHO 2011 https://www.nia.nih.gov/research/publication/global-health-and-aging/preface. Accessed 07 Jul 2016.

[2] Roderick P, Raleigh V, Hallam L, Mallick N (1996). The need and demand for renal replacement therapy in ethnic minorities in England. J Epidemiol Community Health.

[3] Randhawa G (2012). Renal health and transplantation: a focus on ethnicity. J Ren Care.

[4] Coleman D (2013). Immigration, population and ethnicity: the UK in International Perspective.

[5] United Nations Economic and Social Affairs 2013 Inequality Matters. Report on the world social situation 2013 http://www.un.org/esa/socdev/documents/reports/InequalityMatters.pdf. Accessed 07 Jul 2016.

[6] Randhawa G. Tackling health inequalities for minority ethnic groups: challenges and opportunities. A Race Equalities Foundation Briefing Paper 6. 2007. http://www.better-health.org.uk/sites/default/files/briefings/downloads/health-brief6.pdf. Accessed 07 Jul 2016.

[7] Gunaratnam Y. Improving the quality of palliative care – A Race Equality Foundation Briefing Paper. 2007. http://www.better-health.org.uk/sites/default/files/briefings/downloads/health-brief1.pdf. Accessed 07 Jul 2016.

[8] Worth A (2009). Vulnerability and access to care for South Asian Sikh and Muslim patients with life limiting illness in Scotland: prospective longitudinal qualitative study. BMJ.

[9] Gunda S, Thomas M, Smith S (2005). National survey of palliative care in end-stage renal disease in the UK. Nephrolo Dial Transplant.

[10] National Council for Palliative Care 2006. End of Life Care Strategy http://www.ncpc.org.uk/sites/default/files/NCPC_EoLC_Submission.pdf. Accessed 07 Jul 2016.

[11] NHS Kidney Care. National End of Life Care Programme. 2009. End of Life Care in Advanced Kidney Disease: A Framework for Implementation. http://www.ncpc.org.uk/sites/default/files/EndOfLifeCareInAdvancedKidneyDisease.pdf. Accessed 07 Jul 2016.

[12] Morton RL, Tong A, Howard K, Snelling P, Webster AC. The views of patients and carers in treatment decision making for chronic kidney disease: systematic review and thematic synthesis of qualitative studies. Br Med J. http://www.bmj.com/content/bmj/340/bmj.c112.full.pdf. Accessed 07 Jul 2016. 10.1136/bmj.c112 PMC2808468 20085970

[13] Somerville JE (1998). Palliative care: the experience of informal carers within the Bangladeshi community (research abstract). Palliat Med.

[14] Hockley J, Froggatt K, Heimer K. Participatory research in palliative care: Actions and reflections. Oxford University Press: Oxford, UK; 2013. p. 3–14.

[15] Schein E. Clinical Inquiry/Research in The handbook of action research Eds: Reason P, Bradbury H. Sage Publications: London, UK; 2006. p. 185–194.

[16] Simpson L. Does Britain have plural cities? Dynamics of diversity: evidence from the 2011 census ESRC Centre on Dynamics of Ethnicity. 2013; http://www.ethnicity.ac.uk/census/869_CCSR_Bulletin_Does_Britain_have_plural_cities_v7.pdf. Accessed 07 Jul 2016.

[17] Ealing Council 2012 Joint Strategic Needs Assessment Chapter 2 Population Statists and Change. http://www.ealing.gov.uk/downloads/download/1018/ealing_joint_strategic_needs_assessment. Accessed 07 Jul 2016.

[18] Ritchie J, Spencer L, O’Connor W, Ritchie J, Lewis J (2010). Carrying out qualitative analysis. Qualitative research practice: a guide for social science students and researchers.

[19] Wilkinson E, Randhawa G, Brown E, Da Silva Gane M, Stoves J, Warwick G, Atkhar T, Magee R, Sharman S, Farrington K (2014). Communication as care: an emerging issue from an exploratory study of renal end of life care for ethnic minorities in the UK. J Ren Care.

[20] Van Reit Papp J, Mariani E, Chattat R, Koopmans R, Kerherne H, Leppert W, Forycka M, Radbruch L (2015). Identification of the palliative phase in people with dementia: a variety of opinions between healthcare professionals. BMC Palliat Care.

[21] Da Silva Gane M, Braun A, Stott D, Wellsted D, Farrington K (2013). How robust is the ‘surprise question’ in predicting short-term mortality risk in haemodialysis patients?. Nephron Clin Pract.

[22] Murtagh F, Sheerin N, Addinton-Hall J, Higginson I (2011). Trajectories of illness in stage 5 chronic kidney disease: a longitudinal study of patient symptoms and concerns in the last year of life. Clin J Am Soc Nephrol.

[23] Davison S (2010). End of life preferences and needs: perceptions of patients with chronic kidney disease. Clin J Am Soc Nephrol.

[24] Chandna S, Da Silva-Gane M, Marshall C, Warwicker P, Greenwood R, Farrington K (2011). Survival of elderly patients with stage 5 CKD: Comparison of conservative management and renal replacement therapy. Nephrol Dial Transplant.

[25] De Pentheny O’Kelly C, Urch C, Brown E (2011). The impact of culture and religions on truth telling at the end of life. Nephrol Dial Transplant.

[26] Kai J, Beavan J, Faull C (2011). Challenges of mediated communication, disclosure and patient autonomy in cross-cultural cancer care. Br J Cancer.

[27] Bristowe K, Shepherd K, Bryan L, Brown H, Carey I, Matthews B, O’Donoghue D, Vinen K, Murtagh F. The development and piloting of the REnal specific Advanced Communication Training (REACT) programme to improve advance care planning for renal patients. Palliat Med. 2013; doi: 10.1177/1026921631351032. 10.1177/0269216313510342 24201135

[28] Evans N, Menaca A, Koffman J, Haring R, Higginson J, Pool R, Gysels M, on behalf of PRISMA 2012 Cultural competence in end-of-life-care (2012). Terms, definitions, and conceptual models from the British literature. J Palliat Med.

[29] Vickers T, Craig G, Atkin K. Research with black and minority ethnic people using social care services: Methods Review 11 School of Social Care Research. 2012. ISBN 978-0-85328-445-1; http://sscr.nihr.ac.uk/PDF/MR/MR11.pdf

[30] Davison, S. and Simpson, C. Hope and advance care planning in patients with end stage renal disease: qualitative interview study. Br Med J. 2006; doi:10.1136/bmj.38965.626250.55 10.1136/bmj.38965.626250.55 PMC1626305 16990294

[31] Koffman J (2014). Serving multi-cultural needs at the end of life. J Ren Care.

[32] Da Silva Gane M, Farrington K (2014). Supportive care in advanced kidney disease: patient attitudes and expectations. J Ren Care.

